# Association between Ambient Air Pollution and Emergency Room Visits for Respiratory Diseases in Spring Dust Storm Season in Lanzhou, China

**DOI:** 10.3390/ijerph13060613

**Published:** 2016-06-21

**Authors:** Yuxia Ma, Bingshuang Xiao, Chang Liu, Yuxin Zhao, Xiaodong Zheng

**Affiliations:** College of Atmospheric Sciences, Key Laboratory of Semi-Arid Climate Change, Ministry of Education, Center for Meteorological Environment and Human Health, Lanzhou University, Lanzhou 730000, China; xiaobsh14@lzu.edu.cn (B.X.); liuch14@lzu.edu.cn (C.L.); zhaoyx15@lzu.edu.cn (Y.Z.); zhengxd15@lzu.edu.cn (X.Z.)

**Keywords:** air pollutants, emergency room visits, respiratory system diseases, time-series, spring dust storm

## Abstract

*Background*: Air pollution has become a major global public health problem. A number of studies have confirmed the association between air pollutants and emergency room (ER) visits for respiratory diseases in developed countries and some Asian countries, but little evidence has been seen in Western China. This study aims to concentrate on this region. *Methods*: A time-series analysis was used to examine the specific effects of major air pollutants (PM_10_, SO_2_ and NO_2_) on ER visits for respiratory diseases from 2007 to 2011 in the severely polluted city of Lanzhou. We examined the effects of air pollutants for stratified groups by age and gender, accounting for the modifying effect of dust storms in spring to test the possible interaction. *Results*: Significant associations were found between outdoor air pollution concentrations and respiratory diseases, as expressed by daily ER visits in Lanzhou in the spring dust season. The association between air pollution and ER visits appeared to be more evident on dust days than non-dust days. Relative risks (RRs) and 95% CIs per 10 µg/m^3^ increase in 3-day PM_10_ (L3), 5-day SO_2_ (L5), and the average of current and previous 2-day NO_2_ (L01) were 1.140 (1.071–1.214), 1.080 (0.967–1.205), and 1.298 (1.158–1.454), respectively, on dust days. More significant associations between PM_10_, SO_2_ and NO_2_ and ER visits were found on dust days for elderly females, elderly males and adult males, respectively. *Conclusions*: This study strengthens the evidence of dust-exacerbated ER visits for respiratory diseases in Lanzhou.

## 1. Introduction

Ambient air pollution is a global public health concern that is estimated to cause approximately 3.7 million premature deaths per year worldwide [1]. Levels of outdoor air pollution in China are among the highest in the world [2]. Previous studies have showed the strong association of acute effects between particulate matter and morbidity and mortality, mostly as a result of respiratory and cardiovascular diseases [3,4,5,6].

In the last decade, a number of time-series studies have confirmed that air pollution is associated with increases in hospital admissions, mortality, and visits to both general practitioners and emergency rooms in Europe [7,8,9,10], the United States [11,12,13] and Asia [14]. In China, the largest developing country in the world, the relationship between air pollution and daily mortality and morbidity has been examined in several large cities, including Beijing [15,16,17], Shanghai [6,18], and Guangzhou [19]. A recent multi-city analysis including Hong Kong provides evidence supporting the significant acute effect of ambient pollutants on health [20]. A study in Minqin, China reported increased outpatient clinic visits due to respiratory diseases during dust events [21]. Significantly negative correlations between peak expiratory flow rate (PEFR) of children and PM_10_ on the day and three days after a dust event were found in Inner Mongolia [22]. Similar findings were reported for adult population in the Hexi Corridor located upstream from Lanzhou [23,24]. Although most studies have focused on the association of air pollutants with morbidity/mortality from respiratory or cardiovascular diseases, very few analyses used hospital emergency room (ER) visits as the evaluated variable, which may be a more sensitive indicator of the effects of ambient air pollution. 

Lanzhou (E 103°53’ longitude, N 36°03’ latitude), a heavily-polluted industrial city in Western China and the capital city of Gansu Province, is located in the transport pathway of Asian dust storms, severely affected by dust intruding from upwind regions and additional local emissions [25,26,27]. Our study area is limited to the urban districts of Lanzhou (1632 km^2^), a city with a population of 2.90 million in 2013. Lanzhou has a typical temperate, semi-arid continental monsoon climate with four distinctive seasons. Winter is very dry and cold, and summer is sunny and hot. Dust storms occur most frequently in spring.

The aim of this paper is to confirm the acute effect of major air pollutants, including sulfur dioxide (SO_2_), nitrogen dioxide (NO_2_) and particulate matter less than 10 microns in diameter (PM_10_), on ER visits for respiratory diseases among people in Lanzhou in the spring dust storm season during the period 2007–2011. The study examined the associations within overall and stratified groups by gender and age using time-series analyses. The modifying effects of dust *vs.* non-dust days in the association between the three air pollutants and ER visits were analyzed to test whether dust days modify the effect of air pollution on ER visits. Better understanding the adverse effect of outdoor air pollution on ER visits will provide relevant information for developing public health plans and risk assessments in the region.

## 2. Materials and Methods

### 2.1. Data Collection

Data on daily ER visits were collected between 1 March 2007 and 31 May 2011 from three large-scale comprehensive top-level hospitals in Lanzhou, representing the majority of ER visits in Lanzhou. These hospitals have good reputations for treatment of respiratory diseases. In general, over 70% of local residents will choose these hospitals for diagnosis and treatment. There are four comprehensive hospitals in Lanzhou, the other one is a military hospital specialized in orthopedic diseases, brain tumors and Traditional Chinese Medicine, and was therefore excluded from our study along with other smaller specialized hospitals. The cases were extracted and coded according to the International Classification of Disease, tenth revision (ICD-10) for total diseases (ICD: A00-R99) and respiratory (ICD 10: J00-J99). The primary diagnoses were used in this study, as well as age, gender, residential address, date of admission and diagnostic codes. According to the residential addresses, only patients who lived in Lanzhou were included in this study.

Meteorological data, including daily mean temperature, relative humidity, air pressure, visibility, and inversion height, were obtained from the Gansu Meteorological Bureau. The monitoring standard is consistent with international World Meteorological Organization (WMO) standard, and the data are representative, though small variations due to the urban micro-climate effect in parts of the study areas cannot be ruled out. 

Air quality data were provided by Lanzhou Environmental Protection Monitoring Center. The center reports daily observations to the China Environmental Monitoring Chief Station. It is also a part of the nationwide network of monitoring stations. The daily ambient air concentrations of PM_10_, SO_2_ and NO_2_ were provided as daily mean values measured from four fixed monitoring stations located in the urban district of Lanzhou. According to the technical guidelines of the Chinese government, the location of the monitoring station must not be in the direct vicinity of traffic intersections or major industrial polluters and must also have sufficient distance from any other emitting source. Thus the monitoring data adequately reflect the general background urban air pollution level in our study area.

### 2.2. Statistical Methods

As the daily ER visits data represent a small probability event and have a Poisson distribution [28], a Poisson generalized additive model (GAM) approach was used to investigate the associations between daily mean air pollutant concentrations and daily ER visits. Spearman’s correlation coefficients were used to evaluate the inter-relations between air pollutants and weather conditions. There were two steps in the model building and fit. First, an independent model was set up to explore the patterns of the relationship between major air pollutants and the ER visits in the spring dust storm season. Springtime in Lanzhou is defined as from 1 March to 31 May. In general, dust events are defined in terms of dust intensity and visibility, including dust storms, blowing dust and floating dust. Dust storms reduce the horizontal visibility to less than 1000 m, blowing dust reduces the horizontal visibility to 1000–10,000 m, and floating dust reduces the horizontal visibility to less than 10,000 m [29]. If there is a dust event in spring in Lanzhou, it is defined as a dust day. A regressing spline function was used to control for long-term trends and seasonal patterns, as well as the daily mean temperature and relative humidity [30]. The partial autocorrelation function (PACF) was used to select the degrees of freedom of time trends until the absolute values of the sum of PACF for lags up to 30 reached a minimum. Specifically, the degrees of freedom (*df*) per year for time trends were defined for total ER visits (*df* = 6) and respiratory ER visits (*df* = 6). For weather conditions, the selection of degrees of freedom was based on minimizing Akaike’s Information Criterion (AIC) [31]. Residuals of the basic models were used to check whether there were discernible patterns and autocorrelation by means of residual and PACF plots [6]. When the absolute magnitude of the PACF plot was larger than 0.1 for the first two lag days, the core models were added with a regressive term of ER visits on lag 1 day to remove the autocorrelation of residuals, in order to control for potential confounding effects [32]. The independent model 1 is described below:

(1) log[E(Yt)]=α+s(time,df)+DOW+Holiday+s(temperature,df)+s(humidity,df)+βZt

where *t* refers to the day of the observation, *E*(*Yt*) denotes estimated daily ER visits counted on day *t*, α is the intercept, *s*() denotes a regression spline function for nonlinear variables, time is the days of calendar time on day *t*, *df* is the degree of freedom, *DOW* is the day of the week on day *t* (dummy variable), *β* represents the log-relative rate of daily ER visits associated with a unit increase of air pollutants, and *Zt* represents major air pollutant concentrations on day *t*.

After the core models were established, we included the three pollutants in regression models to analyze the relationships between these pollutants and daily ER visits. As the three pollutants were inter-correlated, these pollutants have been included one by one each time to assess the relative risk (RR) posed by each pollutant. Additionally, the sex and age (0–16 years, 16–40 years, 40–60 years, ≥60 years) specific associations between air pollution and ER visits were evaluated. Both single-pollutant models and multiple-pollutant models were fitted with a different combination of pollutants to assess the stability of the major air pollutant influences on ER visits. Delayed effects were considered to investigate single day lags (from L0 to L7) and cumulative day lags (L01 and L07) for air pollutants. A lag of 0 day (L0) corresponds to the current-day air pollutant concentration and a lag of 1 day refers to the previous-day’s air pollutant concentration. Cumulative lags were calculated with the method of averaging the pollutants’ concentration for current and previous days. For example, L02 corresponds to the average of pollutants’ concentration for the current and previous 2 days. Each lag was fitted in the model one at a time. We chose the lagged days with the smallest AIC in model (1) to analyze the other steps in the study. The estimated effects were expressed as relative risks (RRs) with 95% confidence intervals (95% CIs) of the daily ER visits corresponding to a 10 µg/m^3^ increase in air pollutants’ concentrations. Values of *p* < 0.05 were considered statistically significant. All analyses were conducted in R 3.1.3 [33] statistical software (GNU General License, Boston, MA, USA) by using the mgcv package [34,35]. 

## 3. Results

Summary statistics of meteorological variables, air pollutant concentrations and ER visits in spring are shown in Table 1. There were a total number of 41,837 ER visits recorded in spring (460 days, dust days: 32 days, non-dust days: 428 days) from 2007 to 2011. Respiratory ER visits were 30,650, accounting for 73.3% of the total. On average, there were approximately 92 ER visits per day in spring in our study area, among which 67 were due to respiratory diseases. The ratio of male to female respiratory ER visits was 1 to 0.817. During the study period, the mean daily average temperature and humidity were 12.9 °C and 26.8%, reflecting the warm continental monsoon climate of Lanzhou. Meanwhile, the mean daily average pollutant concentrations were 159.2 µg/m^3^ for PM_10_, 45.0 µg/m^3^ for SO_2_, and 42.7 µg/m^3^ for NO_2_. On dust days, the values of PM_10_, SO_2_ and NO_2_ were 324.1 µg/m^3^, 54.0 µg/m^3^ and 46.0 µg/m^3^, respectively. The daily average concentrations of PM_10_ and NO_2_ were higher than the Grade II national air quality limits (70 µg/m^3^ for PM_10_, 60 µg/m^3^ for SO_2_ and 40 µg/m^3^ for NO_2_) (Table 1) on both non-dust days and dust days, especially PM_10_.

The Spearman correlation analysis showed that PM_10_, SO_2_ and NO_2_ had positive correlations with each other, especially between SO_2_ and NO_2_ (*r* = 0.429, *p* < 0.01), and PM_10_ and SO_2_ (*r* = 0.141, *p* < 0.01), whereas the correlation between PM_10_ and NO_2_ was very low (*r* = 0.013, *p* > 0.05) in spring. Meanwhile, pollutant levels were negatively correlated with temperature and relative humidity except PM_10_, which was positively correlated with temperature.

**Figure 1:**
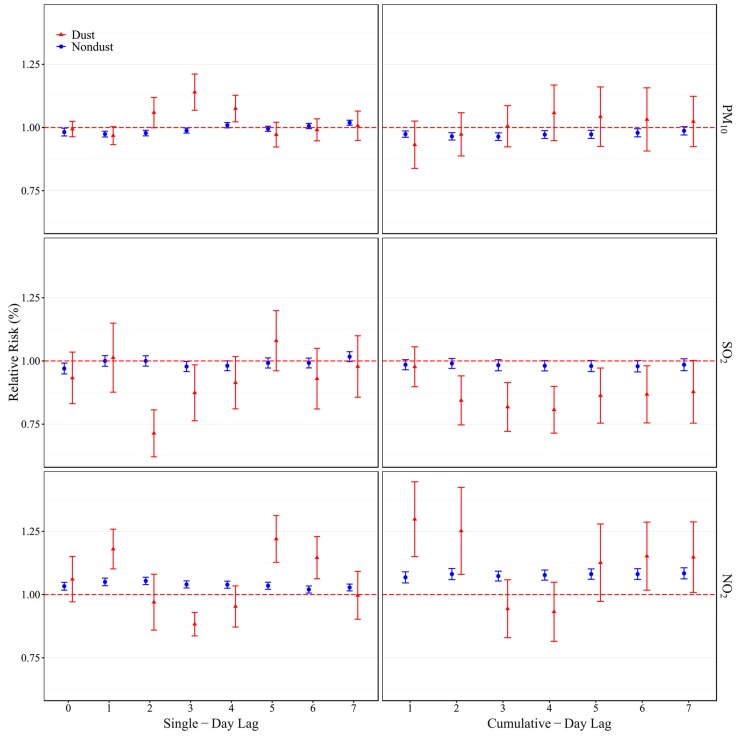
RRs (95%CIs) of ER visits with an increase of 10 µg/m^3^ in air pollutants at single-day lags (**left**) and cumulative-day lags (**right**) in spring in Lanzhou, 2007–2011.

Figure 1 shows the RRs (95% CIs) for ER visits associated with every 10 µg/m^3^ increase in pollutant’s concentrations for different lag structures (single-day lags and cumulative-day lags) after adjustment for the long-term trend, DOW, holiday and weather conditions (RRs and 95% CIs corresponding to the Figure are reported in Table S1). On non-dust days, the greatest RR for PM_10_ and SO_2_ occurred at a 7-day lag (L7), and for NO_2_ at a 2-day lag (L2) in single-day lags, and the greatest RR for PM_10_ reached at lag 07 day (L07), SO_2_ at lag 02 day (L02), and for NO_2_ at lag 07 day (L07) in cumulative-day lags. On dust days, the influence of PM_10_ peaked at a 3-day lag (L3), and the influences of SO_2_ and NO_2_ reached a maximum at a 5-day lag (L5) in single-day lags. Meanwhile, the influence of PM_10_ reached a maximum at lag 04 day (L04), for SO_2_ and NO_2_ at lag 01 day (L01) in cumulative-day lags.

**Figure 2:**
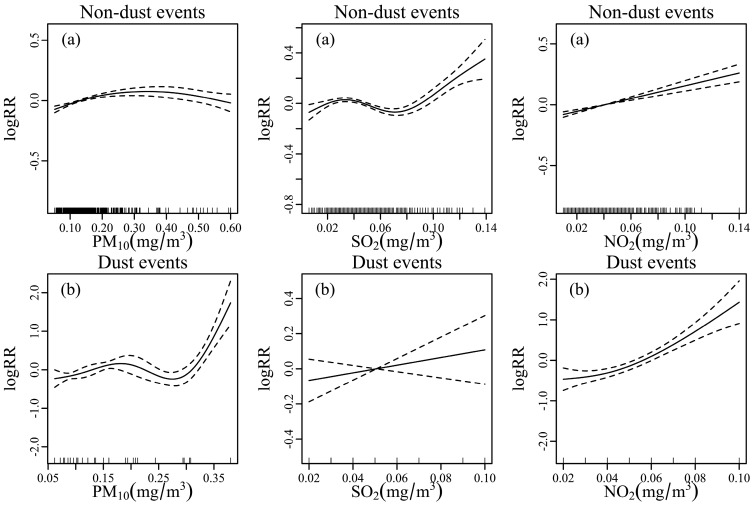
Smoothing plots of air pollutant concentrations against ER visits risk of respiratory under non-dust days (**a**) and dust days (**b**) for PM_10_ (**left**), SO_2_ (**center**), and NO_2_ (**right**). X-axis is the pollutant concentration (mg/m^3^). The solid lines indicate the log RR of ER visits, and the dotted lines represent 95% confidence intervals. Single day lags (L7 for PM_10_ and SO_2_, L2 for NO_2_) were used on non-dust days. Single day lags (L3 for PM_10_, L5 for SO_2_ and NO_2_) were used on dust days. All models were controlled for time trend, DOW, holiday and weather conditions.

Figure 2 shows the exposure-response relationships between air pollutants and ER visits for respiratory diseases during dust events and non-dust events in the single-models. There were similar positive linear relationships between concentration of air pollutants and the respiratory ER visits. Compared with ER visits on non-dust events, visits during dust events for respiratory diseases increased very quickly with the increase of PM_10_, SO_2_ and NO_2_, indicating that the relative risk of ER visits increased with the increase of air pollutant concentrations (particularly particulates) in Lanzhou during the study period.

**Figure 3:**
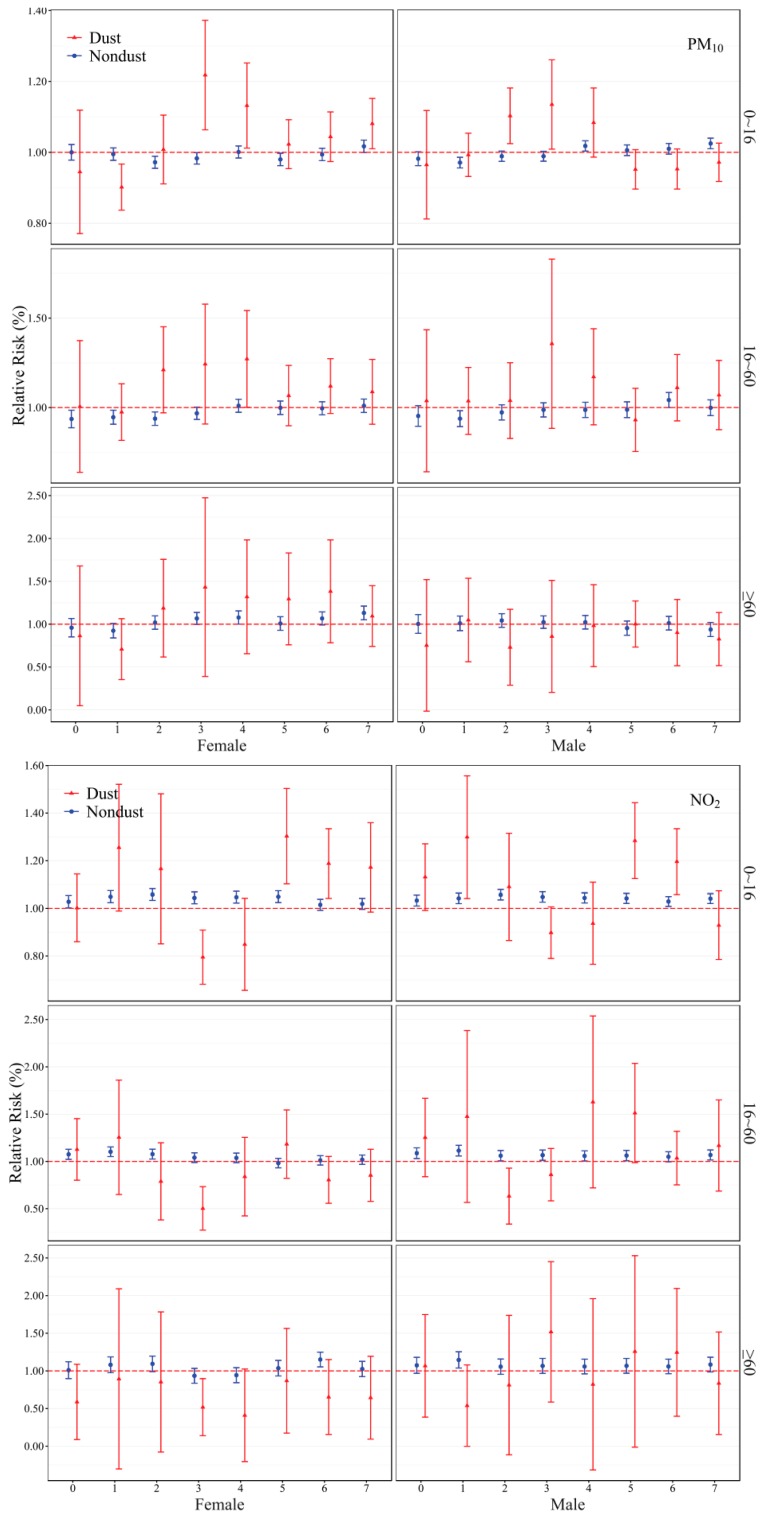

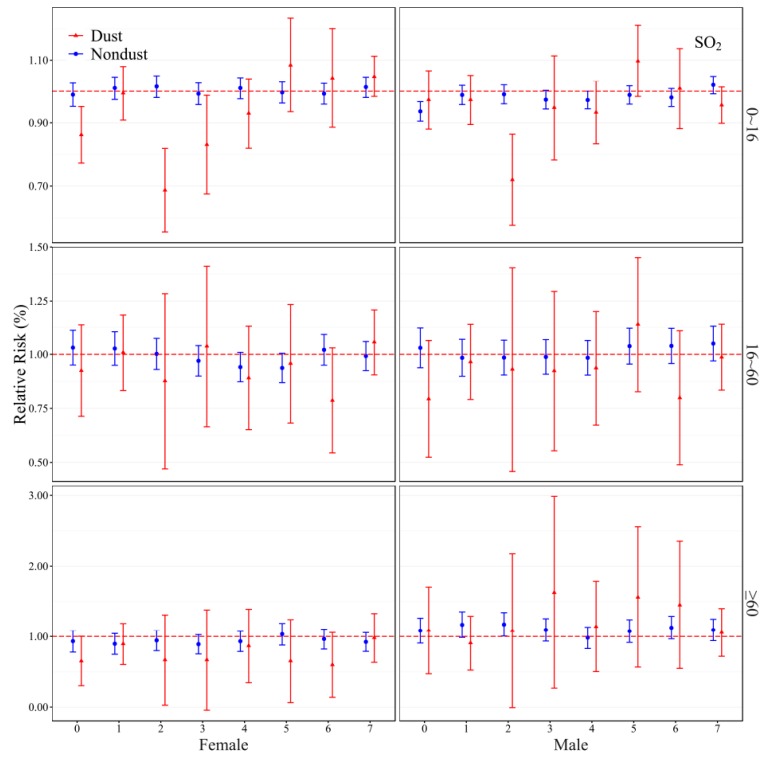
RRs (95% CIs) of ER visits with an increase of 10 µg/m^3^ in air pollutants in multi-pollutant models in spring, 2007–2011. All models were controlled for time trend, DOW, holiday and weather conditions.

Figure 3 shows the sex and age specific influences of air pollutants on ER visits in multi-pollutant models (RRs and 95% CIs corresponding to the Figure are reported in Table S2). Significant associations were found; for example, on non-dust days, the influence of PM_10_ at lag 7 in elderly (≥60) females was significantly greater than other groups, and the influences of NO_2_ at lag 6 in elderly females and lag 1 in elderly males were greater than other groups, while the influence of SO_2_ at lag 2 in elderly males was greater than other groups. On dust days, the influences of PM_10_ at lag 3 in elderly females and lag 3 in adult males was significantly greater than other groups, and the influences of NO_2_ at lag 4 in adult males and lag 3 in elderly males were significantly greater than other groups, while the influence of SO_2_ at lag 3 in elderly males and lag 5 in adult males was significantly greater than other groups.

Table 2 shows the analysis of single-pollutant models and multiple-pollutant models. On non-dust days, the effect of PM_10_ was reduced but remained significant after adjusting for other pollutants, while the effect of NO_2_ increased and remained significant after adjusting for other pollutants. On dust days, the effects of PM_10_ and NO_2_ were reduced but remained significant after adjusting other pollutants. No significant effect was found for SO_2_ both on non-dust and dust days. The effects of PM_10_ and NO_2_ were much higher on dust days than non-dust days. RRs (95% CIs) of ER visits per 10 µg/m^3^ increase in PM_10_ and NO_2_ in multi-pollutant models were 1.084 (1.01–1.16) and 1.150 (1.07–1.24) respectively on dust days.

## 4. Discussion

In this study, we found a significant association between air pollutants and ER visits for respiratory diseases in Lanzhou in the spring dust season during 2007–2011. The association between air pollution and ER visits was more evident on dust days than non-dust days. On dust days, a 10 µg/m^3^ increase in PM_10_, SO_2_ and NO_2_ resulted in 1.6%, 2.7% and 11.0% increase in ER visits in spring, respectively. On non-dust days, a 10 µg/m^3^ increase in PM_10_, SO_2_ and NO_2_ resulted in 0.2%, 0.6% and 2.5% increase in ER visits, respectively. The percentage increases in ER visits on dust days were much higher than that from 2001 to 2005 in Lanzhou. During 2001–2005, respiratory disease hospitalizations increased by 0.2%, 0.55% and 1.1% for a 10 µg/m^3^ increase in PM_10_ at lag 4, SO_2_ and NO_2_ at lag 1, respectively [36]. This matched the fact that dust-exacerbated respiratory diseases increased in spring. These findings provide scientific evidence for local health officials and stakeholders to make decisions about what measures should be taken to protect residents’ well-being in Lanzhou.

The effects of air pollutants were obviously different on non-dust days and dust days. On non-dust days, elderly females (≥60 year of age) appeared to be more vulnerable to PM_10_ and NO_2_ than other groups, while the effect of SO_2_ was found to be strong for elderly males. On dust days, the influence of PM_10_ on ER visits of elderly females and adult males showed much greater associations than other groups. The influence of NO_2_ on adult and elderly males were greater than other groups, and the influence of SO_2_ on elderly males was much greater than other groups. In our study, occupation data is not available, so we cannot take into consideration occupational exposure. Adults (students, employees and outdoor workers) being more affected by air pollution might be explained by their higher exposure to particulate pollutant on dust days. Thus, this age group has a higher exposure risk than the others. The elderly were considered susceptible groups and have a lower ability to regulate their bodies when affected by air pollution compared with adults, so they were impacted by diseases much more easily. Elderly (≥60) people are usually susceptible to air pollution as the high-risk group compared with the younger people [37], especially for those patients with respiratory diseases who may not adjust well to serious air pollution episodes in spring.

Interestingly, we found a greater effect of ambient air pollution on ER visits for total females than males on non-dust days. This is consistent with the results from a previous study that suggested that females were more vulnerable to outdoor air pollution [18]. Reasons for our sex-specific observations are unclear and require further investigation. Females have much lower smoking rate than males (0.6% *vs.* 50.6%) [38]. One study suggested that effects of air pollution may be stronger in nonsmokers than in smokers [39]. Oxidative and inflammatory effects of smoking may desensitize males to additional exposure to air pollutants that may not further enhance effects along the same pathways. But on dust days, the effects of PM_10_ SO_2_ and NO_2_ were greater in total males than in females. This could be explained by males’ potential higher exposure to air pollutants because outside workers are usually males in Lanzhou. In addition, women frequently wear face masks to cope with the cold weather and pollutants, which may also possibly protect them during the most severe episodes.

The strongest association between air pollutant and daily ER visits for respiratory diseases occurs at a 3-day lag (L3) for PM_10_, 5-day lag (L5) for SO_2_ and 1-day lag (L01) for NO_2_ on dust days. Particulate pollution is mainly caused by fossil-fuel combustion, local heavy industrial emission, and remote transport of dust storms to Lanzhou. During the dust season, particulate pollution is mostly attributed to dust storms, and the daily concentrations of PM_>10_ and PM_2.5__–10_ increased to 1.3 times and 9.5 times of the values before dust events, respectively [40]. Our findings confirmed those of earlier large analyses in Europe [41,42] and Asia [43,44] on dust-exacerbated respiratory diseases in children and increased hospitalization. Previous studies suggested that health effects may be related more to PM specific components rather than mass, although it is still uncertain whether the health problems are mainly related to PM physical characteristics or to PM chemical characteristics [45]. Previous studies reported that the high concentration of PM_10_ in Lanzhou was mainly related to the intrusion of dust events from the upwind, the industrial and domestic emissions, and the secondary dust entrainment due to the local increasing wind speeds during dust events [26]. The chemical elements of TSPs during the dust storm period in Lanzhou were characterized by elements such as Si, Al, K, Ca, Ti, Mg, Na, Fe, S, O and C [26]. Al, Si, S, Cl, K, Ca, Ti, Cr, Mn, Fe and Cu were identified in the inhalable particulate matter [46].

SO_2_ is a gaseous pollutant mainly emitted by fuel combustion and has been found to be significantly associated with increased respiratory problems [16,47]. Previous research also showed that an increase of 10 µg/m^3^ of SO_2_ corresponded to a 1.8% increase in total mortality and a 3.2% increase of respiratory mortality [48]. In the Hong Kong intervention study, a decrease of 10 µg/m^3^ in SO_2_ was associated with a 1.1% decrease of total mortality and a 2.0% decrease of respiratory mortality [49]. NO_2_ is a ubiquitous gaseous pollutant which contributes to respiratory inflammation. Motor vehicle exhaust and industrial emissions are the major anthropogenic sources of NO_2_ in Lanzhou [26]. Multi-city analyses conducted in Europe [50,51] and Canada [52] provide further evidence supporting the short-term association between NO_2_ and increased mortality risk. The increased health impact of two gaseous pollutants on respiratory diseases also has been widely reported, mainly in urban areas of China [48,53]. In addition, the three pollutants were inter-correlated, and greater effects of SO_2_ and NO_2_ that we observed during the dust season might also be due to PM_10_, which was higher during that season. In fact, coal combustion is the major source of both particulate and gaseous pollutants in China since the 1990s, thus limiting our ability to separate the independent effect for the individual pollutants.

In general, air pollutants can oxidize mitochondria and cause apoptosis or necrosis of macrophages and respiratory epithelial cells, and then possibly decrease the host defense to respiratory infection or increasing airway reactivity [54]. Also, patients with respiratory disease (e.g., COPD) often have a systemic deficit in their antioxidant defenses, and air pollution could produce a significant additive oxidative stress as a response to an inflammation of the lungs [55].

In the multiple models, we also found statistically significant associations between air pollutants and respiratory ER visits both on non-dust days and dust days, but effects of three pollutants were much greater on dust days than non-dust days. This result helps confirm the health effects of air pollution caused by dust storms [21,22,23,24]. The Pearson correlation coefficients among those pollutants vary in different regions, and the results in spring in Lanzhou for this study (between SO_2_ and NO_2_ (*r* = 0.429), between PM_10_ and SO_2_ (*r* = 0.141), and between PM_10_ and NO_2_ (*r* = 0.013)) were lower than those in Europe [8,9], America [56], and other cities in China [57,58]. The correlation coefficients ranged from 0.39 to 0.72 during the period of 2001–2005 in Lanzhou [36]. A study in the Republic of Korea showed that the correlation coefficients ranged from 0.44 to 0.72 for the entire year and from 0.04 to 0.38 in spring [59]. Similar results about the correlation coefficients were found in this study, yet no report about the reasons for the regional differences in these correlation coefficients has been documented. During the dust storm season in spring, it might be because fine particles are diffused by strong wind in the dust storm events [40]. Our findings of significant associations between air pollutants and respiratory ER visits during the spring dust season is, in part, consistent with previous results of stronger associations between ER visits and pollutants during the cold season [15,16]. In previous studies, the cold season (from November to April) included most of the dust season (from March to May) in this study. Therefore, the spring dust season could be considered as somewhat of a surrogate to the cool season.

Our findings of the stronger effects of air pollution in spring might be attributed to the special topography of the narrow but long NW-SE oriented valley basin of the Qinghai-Tibet Plateau. Due to the special topographic characteristics, the frequencies of both calm wind and thermal inversion in the atmospheric boundary layer are very high [40]. Even in December, the monthly frequency of calm wind reaches as high as 92.6% [60]. Lanzhou is also located in the transport pathway of Asian dust storms. Therefore, a large input of desert dust from the upwind region in spring is an important factor in the serious air pollution in Lanzhou. Meanwhile, coal is the main source for household heating in winter, providing more than 70% of the total energy in Lanzhou [61]. Some limitations in our study should be noted. Hospital visits and pollution data we collected were limited to three hospitals and four monitoring stations, and thus selection biases may exist. Additionally, the observed health effects of SO_2_ and NO_2_ in our study might also be related to exposures to fine particles or traffic-related emissions. However, due to lack of available information on personal exposure in China, we could not quantify these biases.

## 5. Conclusions

In summary, we found ambient air pollution was associated with ER visits for respiratory diseases within the general population of Lanzhou, China. Our results suggest that the spring dust season and socio-demographic factors (e.g., gender, age) may modify the acute health effects of air pollution. Further studies with accurate exposure measurement are needed to evaluate the precise magnitudes of morbidity effects of ambient air pollutants. Exposure measurement error has been cited as a major limitation in such studies [62,63]. The results of our present study provide new information about the effects of air pollution in developing countries and may have implications for local environmental and social policies.

## Figures and Tables

**Table 1:** Descriptive statistics on meteorological variables, air pollution levels and ER visits in spring time in Lanzhou, 2007–2011.

Daily Data	Mean	SD	Min	P25	Median	P75	Max
***Metrologic measures***							
Temperature (°C)	12.9	6.2	−5.1	8.3	13.6	17.9	24.8
Relative humidity (%)	26.8	14.8	4.0	15.3	23.0	33.0	87.0
***Air pollutants concentrations***							
PM_10_ (µg/m^3^)	159.2	103.0	52.0	94.0	134.0	183.0	600.0
SO_2_ (µg/m^3^)	45.0	22.0	10.0	30.0	40.0	60.0	140.0
NO_2_ (µg/m^3^)	42.7	22.0	10.0	30.0	50.0	50.0	140.0
***Dust days***							
PM_10_ (µg/m^3^)	324.0	178.0	86.0	175.0	268.0	491.0	600.0
SO_2_ (µg/m^3^)	54.0	22.0	14.0	43.0	51.0	61.0	122.0
NO_2_ (µg/m^3^)	46.0	16.0	10.0	37.0	44.0	59.0	78.0
***Non-dust days***							
PM_10_ (µg/m^3^)	146.0	83.0	52.0	92.0	130.0	168.0	600.0
SO_2_ (µg/m^3^)	43.0	22.0	6.0	28.0	39.0	55.0	139.0
NO_2_ (µg/m^3^)	41.0	22.0	11.0	25.0	34.0	51.0	140.0
***ER* visits**							
Total	91.0	47.6	12.0	51.0	83.0	120.0	246.0
Respiratory	66.6	28.4	10.0	44.0	66.0	88.0	165.0

SD: Standard deviation; Min: minimum; P25: 25th percentile; P75: 75th percentile; Max: maximum.

**Table 2:** RRs (95% CIs) per 10 µg/m^3^ increase in PM_10_, SO_2_ and NO_2_ on ER visits in single and multiple pollutant models in spring, 2007–2011 *.

Models/PM_10_	RR (95% CI)	*p*	SO_2_	RR (95% CI)	*p*	NO_2_	RR (95% CI)	*p*
***Non-dust days***								
PM_10_	0.974 (0.96–0.99)	<0.01	SO_2_	0.714 (0.63–0.81)	<0.01	NO_2_	1.054 (1.04–31.07)	<0.01
+SO_2_+NO_2_	0.966 (0.95–0.98)	<0.01	+PM_10_+NO_2_	0.789 (0.70–0.90)	<0.01	+PM_10_+SO_2_	1.068 (1.05–1.08)	<0.01
***Dust days***								
PM_10_	1.140 (1.07–1.21)	<0.01	SO_2_	0.970 (0.95–0.99)	<0.01	NO_2_	1.220 (1.13–1.32)	<0.01
+SO_2_+NO_2_	1.084 (1.01–1.16)	0.018	+PM_10_+NO_2_	0.947 (0.93–0.97)	<0.01	+PM_10_+SO_2_	1.150 (1.07–1.24)	<0.01

* Single day lags (L1 for PM_10_, L0 for SO_2_ and L2 for NO_2_) were used on non-dust days. Single day lags (L3 for PM_10_, L2 for SO_2_ and L5 for NO_2_) were used on dust days. All models were controlled for time trend, DOW, holiday, weather conditions.

## Supplementary Materials

The following are available online at www.mdpi.com/1660-4601/13/6/613/s1, Table S1: RRs (95% CIs) of ER visits with an increase of 10 µg/m^3^ in air pollutants at single-day lags and cumulative-day lags in spring in Lanzhou, 2007–2011, Table S2: RRs (95% CIs) of ER visits with an increase of 10 µg/m^3^ in air pollutants in multi-pollutant models in spring, 2007–2011.
