# Supplementary material for: Association between Ambient Air Pollution and Emergency Room Visits for Respiratory Diseases in Spring Dust Storm Season in Lanzhou, China
Source: Int J Environ Res Public Health. 2016 Jun 21;13(6):613. doi: 10.3390/ijerph13060613 (PMC4924070; doi:10.3390/ijerph13060613)
Supplement: Supplementary file 1 [file ijerph-13-00613-s001.pdf]

Supplementary Materials: Association between Ambient Air Pollution and Emergency Room Visits for Respiratory Diseases in Spring Dust Storm Season in Lanzhou, China

Yuxia Ma, Bingshuang Xiao, Chang Liu, Yuxin Zhao and Xiaodong Zheng

Table S1. RRs (95% CIs) of ER visits with an increase of 10 μg/m³ in air pollutants at single-day lags and cumulative-day lags in spring in Lanzhou, 2007–2011 *.

Lags	Non-Dust Days		Dust Days	
	RR (95% CI)	*p* Value	RR (95% CI)	*p* Value
PM_{10}	*Single-day lag*			
0	0.982 (0.97, 1.00)	0.025	0.994 (0.96, 1.03)	0.704
1	0.974 (0.96, 0.99)	0.000	0.968 (0.93, 1.00)	0.083
2	0.978 (0.97, 0.99)	0.000	1.059 (1.00, 1.12)	0.045
3	0.988 (0.98, 1.00)	0.000	**1.140 (1.07, 1.21)**	0.000
4	1.009 (1.00, 1.02)	0.099	1.075 (1.02, 1.13)	0.003
5	0.995 (0.99, 1.01)	0.368	0.972 (0.92, 1.02)	0.269
6	1.006 (1.00, 1.02)	0.223	0.991 (0.95, 1.04)	0.677
7	**1.019 (1.01, 1.03)**	0.000	1.007 (0.95, 1.07)	0.809
	Cumulative-day lag			
01	0.974 (0.96, 0.99)	0.000	0.932 (0.84, 1.03)	0.169
02	0.965 (0.95, 0.98)	0.000	0.973 (0.89, 1.06)	0.537
03	0.964 (0.95, 0.98)	0.000	1.005 (0.93, 1.09)	0.901
04	0.972 (0.96, 0.99)	0.001	**1.058 (0.95, 1.17)**	0.283
05	0.973 (0.96, 0.99)	0.001	1.043 (0.93, 1.18)	0.467
06	0.979 (0.96, 1.00)	0.009	1.032 (0.92, 1.17)	0.607
07	**0.987 (0.97, 1.00)**	0.133	1.024 (0.93, 1.13)	0.635
SO_2	*Single-day lag*			
0	0.970 (0.95, 0.99)	0.006	0.933 (0.84, 1.04)	0.212
1	1.000 (0.98, 1.02)	0.998	1.013 (0.89, 1.16)	0.856
2	1.000 (0.98, 1.02)	0.974	0.714 (0.63, 0.81)	0.000
3	0.978 (0.96, 1.00)	0.030	0.874 (0.77, 0.99)	0.037
4	0.981 (0.96, 1.00)	0.057	0.914 (0.82, 1.02)	0.118
5	0.992 (0.97, 1.01)	0.411	**1.080 (0.97, 1.21)**	0.173
6	0.992 (0.97, 1.01)	0.425	0.930 (0.82, 1.06)	0.271
7	**1.017 (1.00, 1.04)**	0.090	0.978 (0.86, 1.11)	0.726
	Cumulative-day lag			
01	0.985 (0.97, 1.01)	0. 134	**0.977 (0.90, 1.06)**	0.568
02	**0.990 (0.97, 1.01)**	0.299	0.844 (0.75, 0.95)	0.004
03	0.983 (0.96, 1.01)	0.128	0.818 (0.73, 0.92)	0.001
04	0.981 (0.96, 1.00)	0.068	0.807 (0.72, 0.90)	0.000
05	0.980 (0.96, 1.00)	0.075	0.863 (0.76, 0.98)	0.022
06	0.979 (0.96, 1.00)	0.076	0.868 (0.76, 0.99)	0.032
07	0.985 (0.96, 1.01)	0.208	0.878 (0.76, 1.01)	0.070
NO_2	*Single-day lag*			
0	1.033 (1.02, 1.05)	0.000	1.061 (0.8, 1.16)	0.170
1	1.050 (1.04, 1.07)	0.000	1.180 (1.10, 1.26)	0.000
2	**1.054 (1.04, 1.07)**	0.000	0.970 (0.87, 1.09)	0.594
3	1.040 (1.03, 1.05)	0.000	0.883 (0.84, 0.93)	0.000
4	1.039 (1.03, 1.05)	0.000	0.953 (0.88, 1.04)	0.272
5	1.035 (1.02, 1.05)	0.000	**1.220 (1.13, 1.32)**	0.000
6	1.020 (1.01, 1.03)	0.005	1.146 (1.07, 1.23)	0.000
7	1.028 (1.01, 1.04)	0.000	0.997 (0.91, 1.10)	0.947

	Cumulative-day lag			
01	1.068 (1.05, 1.09)	0.000	**1.298 (1.16, 1.45)**	0.000
02	1.081 (1.06, 1.10)	0.000	1.252 (1.09, 1.44)	0.001
03	1.073 (1.05, 1.10)	0.000	0.944 (0.84, 1.07)	0.348
04	1.077 (1.058, 1.098)	0.000	0.932 (0.822, 1.056)	0.266
05	1.081 (1.061, 1.102)	0.000	1.126 (0.983, 1.289)	0.087
06	1.081 (1.059, 1.102)	0.000	1.152 (1.025, 1.294)	0.017
07	**1.084 (1.062, 1.106)**	0.000	1.148 (1.017, 1.296)	0.026

* All models were controlled for time trend, DOW, holiday and weather conditions.

Table S2. RRs (95% CIs) of ER visits with an increase of 10 μg/m³ in air pollutants in multi-pollutant models in spring, 2007–2011 *.

**Sex and**	**PM_{10}**		**SO_2**			**NO_2**
**Ages**	**RR (95% CI)**	***p* Value**	**RR (95% CI)**	***p* Value**	**RR (95% CI)**	***p* Value**
**Non-Dust Days**						
**Sex**						
Female	0.968 (0.95, 0.99)	0.000	0.972 (0.94, 1.00)	0.086	1.061 (1.04, 1.08)	0.000
Male	0.958 (0.94, 0.97)	0.000	0.925 (0.90, 0.95)	0.000	1.074 (1.05, 1.10)	0.000
**Age**						
0–16	1.016 (1.01, 1.03)	0.005	**0.943 (0.92, 0.97)**	0.000	1.066 (1.05, 1.08)	0.000
16–40	0.940 (0.91, 0.98)	0.001	0.931 (0.87, 1.00)	0.039	1.117 (1.07, 1.17)	0.000
40–60	**1.051 (1.004, 1.1)**	0.033	0.920 (0.83, 1.01)	0.093	1.106 (1.03, 1.19)	0.004
≥60	1.049 (0.99, 1.10)	0.07	0.915 (0.82, 1.02)	0.121	**1.118 (1.04, 1.20)**	0.003
**Dust days**						
**Sex**						
Female	0.965 (0.91, 1.03)	0.252	0.866 (0.71, 1.06)	0.161	0.871 (0.79, 0.96)	0.004
Male	0.968 (0.90, 1.04)	0.384	0.870 (0.73, 1.04)	0.127	1.208 (1.10, 1.33)	0.000
**Age**						
0–16	1.097 (1.00, 1.20)	0.043	**0.766 (0.67, 0.88)**	0.000	**1.211 (1.10, 1.33)**	0.000
16–40	**1.372 (1.06, 1.78)**	0.018	0.741 (0.55, 1.00)	0.051	0.812 (0.59, 1.11)	0.197
40–60	**1.113 (0.80, 1.54)**	0.524	**0.759 (0.39, 1.48)**	0.416	**1.060 (0.51, 2.19)**	0.875
≥60	1.002 (0.69, 1.46)	0.992	0.575 (0.37, 0.91)	0.017	0.357 (0.12, 1.08)	0.068

* Single day lags (L1 for PM_{10}, L0 for SO_2 and L2 for NO_2) were used for males and single day lags (L2 for PM_{10} and NO_2, and L3 for SO_2) were used for females on non-dust days. Single day lags (L5 for PM_{10} and NO2, L2 for SO_2) were used for males and single day lags (L1 for PM_{10}, and L3 for NO_2 and SO_2) were used for females on dust days. All models were controlled for time trend, DOW, holiday and weather conditions.

© 2016 by the authors; licensee MDPI, Basel, Switzerland. This article is an open access article distributed under the terms and conditions of the Creative Commons by Attribution (CC-BY) license (http://creativecommons.org/licenses/by/4.0/).
